# Cedar Virus: A Novel Henipavirus Isolated from Australian Bats

**DOI:** 10.1371/journal.ppat.1002836

**Published:** 2012-08-02

**Authors:** Glenn A. Marsh, Carol de Jong, Jennifer A. Barr, Mary Tachedjian, Craig Smith, Deborah Middleton, Meng Yu, Shawn Todd, Adam J. Foord, Volker Haring, Jean Payne, Rachel Robinson, Ivano Broz, Gary Crameri, Hume E. Field, Lin-Fa Wang

**Affiliations:** 1 CSIRO Livestock Industries, Australian Animal Health Laboratory, Geelong, Australia; 2 Queensland Centre for Emerging Infectious Diseases, Biosecurity Queensland, Coopers Plain, Australia; Mount Sinai School of Medicine, United States of America

## Abstract

The genus *Henipavirus* in the family *Paramyxoviridae* contains two viruses, Hendra virus (HeV) and Nipah virus (NiV) for which pteropid bats act as the main natural reservoir. Each virus also causes serious and commonly lethal infection of people as well as various species of domestic animals, however little is known about the associated mechanisms of pathogenesis. Here, we report the isolation and characterization of a new paramyxovirus from pteropid bats, Cedar virus (CedPV), which shares significant features with the known henipaviruses. The genome size (18,162 nt) and organization of CedPV is very similar to that of HeV and NiV; its nucleocapsid protein displays antigenic cross-reactivity with henipaviruses; and it uses the same receptor molecule (ephrin- B2) for entry during infection. Preliminary challenge studies with CedPV in ferrets and guinea pigs, both susceptible to infection and disease with known henipaviruses, confirmed virus replication and production of neutralizing antibodies although clinical disease was not observed. In this context, it is interesting to note that the major genetic difference between CedPV and HeV or NiV lies within the coding strategy of the P gene, which is known to play an important role in evading the host innate immune system. Unlike HeV, NiV, and almost all known paramyxoviruses, the CedPV P gene lacks both RNA editing and also the coding capacity for the highly conserved V protein. Preliminary study indicated that CedPV infection of human cells induces a more robust IFN-β response than HeV.

## Author Summary

Hendra and Nipah viruses are 2 highly pathogenic paramyxoviruses that have emerged from bats within the last two decades. Both are capable of causing fatal disease in both humans and many mammal species. Serological and molecular evidence for henipa-like viruses have been reported from numerous locations including Asia and Africa, however, until now no successful isolation of these viruses have been reported. This paper reports the isolation of a novel paramyxovirus, named Cedar virus, from fruit bats in Australia. Full genome sequencing of this virus suggests a close relationship with the henipaviruses. Antibodies to Cedar virus were shown to cross react with, but not cross neutralize Hendra or Nipah virus. Despite this close relationship, when Cedar virus was tested in experimental challenge models in ferrets and guinea pigs, we identified virus replication and generation of neutralizing antibodies, but no clinical disease was observed. As such, this virus provides a useful reference for future reverse genetics experiments to determine the molecular basis of the pathogenicity of the henipaviruses.

## Introduction

Henipaviruses were first discovered in the 1990s following investigation of serious disease outbreaks in horses, pigs and humans in Australia and Malaysia [1], [2] and comprise the only known Biosafety Level 4 (BSL4) agents in the family *Paramyxoviridae* [3]. Depending upon the geographic locations of outbreaks, and the virus and animal species involved, case mortality is between 40% to 100% in both humans and animals [4], [5], making them one of the most deadly group of viruses known to infect humans. The genus *Henipavirus* in the subfamily *Paramyxovirinae* currently contains two members, Hendra virus (HeV) and Nipah virus (NiV) [6]. Fruit bats in the genus *Pteropus*, commonly known as flying foxes, have been identified as the main natural reservoir of both viruses although serological evidence suggests that henipaviruses also circulate in non-pteropid bats [7], [8], [9], [10].

The discovery of henipaviruses had a significant impact on our understanding of genetic diversity, virus evolution and host range of paramyxoviruses. Paramyxoviruses, such as measles virus and canine distemper virus, were traditionally considered to have a narrow host range and to be genetically stable with a close to uniform genome size shared by all members of *Paramyxovirinae* [3]. Henipaviruses shifted this paradigm on both counts having a much wider host range and a significantly larger genome [6]. Identification of bats as the natural reservoir of henipaviruses also played an important role in significantly increasing international scientific attention on bats as an important reservoir of zoonotic viruses, including Ebola, Marburg, SARS and Melaka viruses [11], [12], [13], [14].

Since the discovery of the first henipavirus in 1994, much progress has been made in henipavirus research, from identification of functional cellular receptors to the development of novel diagnostics, vaccine and therapeutics [15], [16], [17], [18], [19], [20], [21], [22], [23], [24], [25]. By contrast, there is little understanding of the pathogenesis of these highly lethal viruses. This is due in part to the requirement of a high security BSL4 facility for any live infection studies and in part to the limited range of research tools and reagents for the current small animal models. Research into the mechanisms of henipavirus pathogenesis is also hampered by the lack of related, but non-pathogenic or less pathogenic viruses, thus preventing targeted comparative pathogenetic studies.

Early serological investigations in Australia and more recent studies in other regions (e.g., China) indicated the presence of cross-reactive, but not cross-neutralizing, antibodies to henipaviruses in bats of different species [8]. These findings were further supported by the detection of henipavirus-like genomic sequences in African bats [26]. Discovery and isolation of these related viruses will be highly important to our further understanding of henipavirus evolution, mechanism of cross-species transmission, and pathogenesis in different animal species.

Here we report the isolation and characterization of a new bat henipavirus which, based on preliminary infection studies, is non-pathogenic in two of the small animal infection models currently used in henipavirus research. We believe that this new virus will provide a powerful tool to facilitate our future study into different aspects of henipaviruses, especially in the less advanced area of pathogenesis.

## Results

### Virus isolation from pooled bat urine samples

As part of our on-going field studies on HeV genetic diversity and infection dynamics in the Queensland flying fox populations, urine samples were collected on a regular basis for PCR and virus isolation. Since the establishment of the *Pteropus alecto* primary cell lines in our group [27], we have intensified our effort to isolate live virus from these urine samples by routinely inoculating separate primary cell lines derived from kidney, spleen, brain, and placenta, as well as Vero cells. Syncytial CPE was observed in kidney cell (PaKi) monolayers 5 days post inoculation (dpi) with two different urine samples (Fig. S1) collected in September 2009 from a flying fox colony in Cedar Grove, South East Queensland (see Fig. S2 for map location). No CPE was observed in any of the four other cell lines. Supernatant harvested 6 dpi was used to inoculate fresh PaKi cell monolayers. After two passages in PaKi cells, the virus was able to infect and cause CPE in Vero cells. However, the CPE morphology of CedPV infection in Vero cells was different from that of HeV infection. Further analysis using HeV-specific PCR primers indicated that the new bat virus was not an isolate of HeV.

### Genome analysis of the newly isolated virus

Considering the formation of syncytial CPE by this new virus and the previous success in isolating paramyxoviruses from bat urine [28], [29], [30], paramyxovirus family-specific and genus-specific primers were used to determine whether this new virus was a member of the family *Paramyxoviridae*. Positive PCR fragments of the expected sizes were obtained from the *Paramyxovirinae* and *Respirovirus/Morbillivirus/Henipavirus* primer sets developed by Tong et al [31]. Sequencing of the PCR products indicated that it was a new paramyxovirus most closely related to HeV and NiV. Based on these preliminary data, the virus was named Cedar virus (CedPV) after the location of the bat colony sampled.

**Figure 1:**
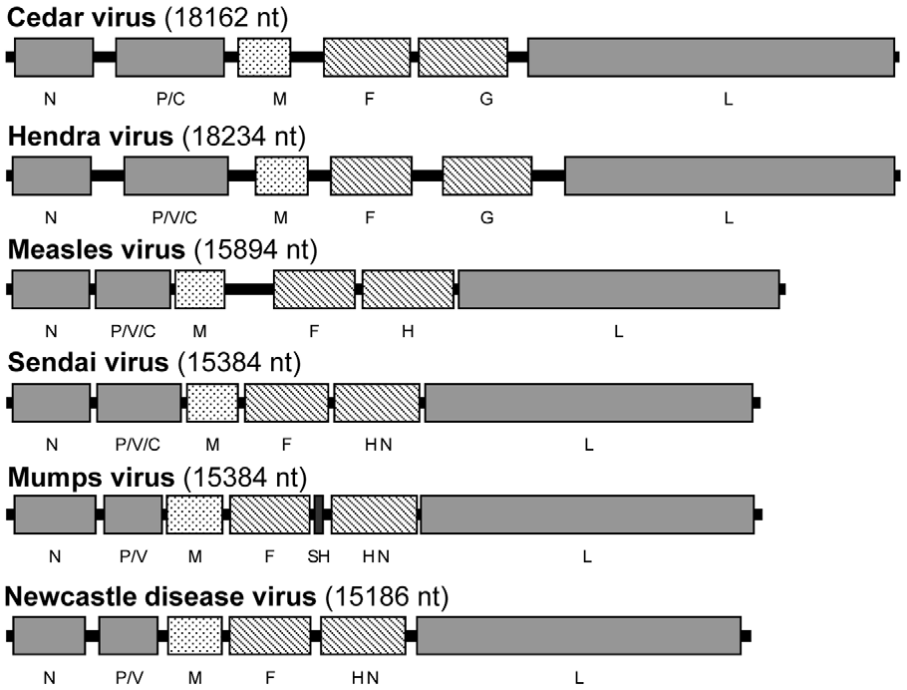
Comparison of genome size and organization of CedPV to those of the prototype viruses of the five existing genera in the subfamily *Paramyxovirinae*. Each of the coding and non-coding regions is drawn to scale. The six major genes present in all paramyxovirus genomes are indicated as follows: light shaded = RNA polymerase and nucleocapsid genes (N, P and L); slanted = envelope membrane protein genes (F and attachment protein); dotted = matrix protein (M). The dark shaded box represents the gene (SH) not commonly shared among members of the subfamily.

Full length genome sequence was determined by a combination of three different approaches, random deep sequencing using 454 technology, sequencing of PCR products obtained using degenerate primers designed based on known henipaviruses, and RACE to determine the precise genome terminal sequences. As shown in Fig. 1, the genome of CedPV is 18,162 nt in length most similar to that of HeV in the family. The full genome sequence has been deposited to GenBank (Accession No. JQ001776). The genome size is a multiple of 6, hence abiding by the Rule-of-Six observed for all known members of the subfamily *Paramyxovirinae* [3]. It has a 3-nt intergenic sequence of CTT absolutely conserved at all seven positions and highly conserved gene start and stop signals similar to those present in HeV and NiV (Fig. S3). Also similar to the HeV genome is the presence of relatively large non-coding regions in the CedPV genome (Fig. 1 and Table 1). The overall protein-coding capacity of the CedPV genome is 87.41% which is significantly lower than the average of 92.00% for other family members but higher than HeV at 82.12%. As the genome size of CedPV and HeV is very similar, the increased coding capacity of CedPV is attributed to an increase in protein sizes for five of the six major proteins, with the L protein being 257-aa larger (Table 1). At 2,501 aa, the CedPV L protein is the largest, not only in the family *Paramyxoviridae* but also for all known viruses in the order Mononegavirale.

**Table 1:** Comparison of common genes among CedPV, HeV and NiV.

Gene	Virus	Open Reading Frame			Length of Untranslated Regions (nt)	
		Length (aa)	% sequence identity to CedPV	% sequence identity to HeV	5′ UTR	3′UTR
N	CedPV	510			88	334
	HeV	532	58		57	568
	NiV	532	59	92	57	586
P	CedPV	737			98	192
	HeV	707	25		105	469
	NiV	709	27	65	105	469
C	CedPV	177				
	HeV	166	26			
	NiV	166	25	83		
M	CedPV	359			114	408
	HeV	352	60		100	200
	NiV	352	60	89	100	200
F	CedPV	557			276	88
	HeV	546	42		272	418
	NiV	546	43	87	284	412
G	CedPV	622			98	139
	HeV	604	29		233	516
	NiV	602	30	78	233	504
L	CedPV	2501			293	63
	HeV	2244	50		153	67
	NiV	2244	50	86	153	67

**Figure 2:**
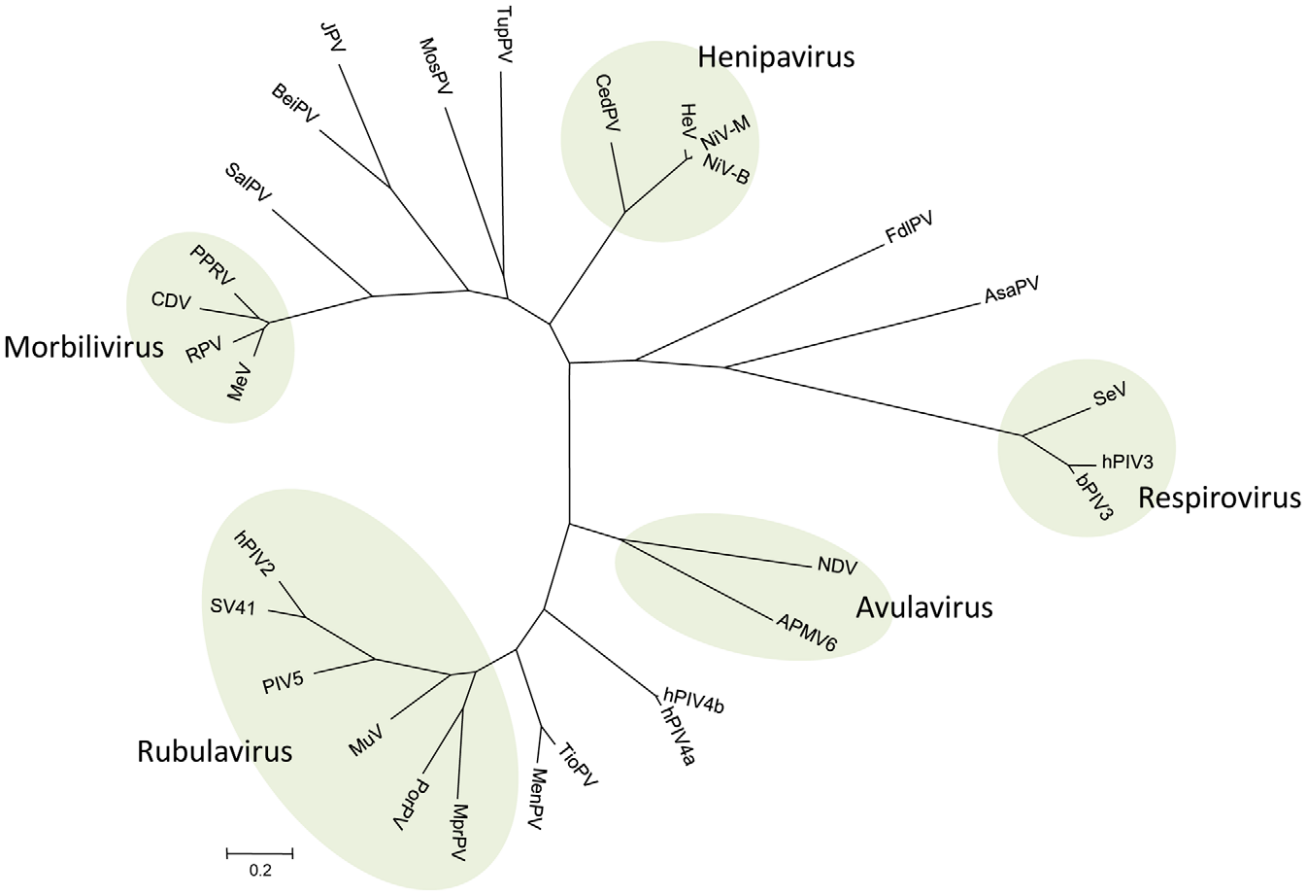
Phylogenetic tree based on the N protein sequences of selected paramyxoviruses. Virus name (abbreviation) and GenBank accession numbers are as follows: Avian paramyxovirus 6 (APMV6) AY029299; Atlantic salmon paramyxovirus (AsaPV) EU156171; Beilong virus (BeiPV) DQ100461; Bovine parainfluenza virus 3 (bPIV3) AF178654; Canine distemper virus (CDV) AF014953; Cedar virus (CedPV) JQ001776; Fer-de-lance virus (FdlPV) AY141760; Hendra virus (HeV) AF017149; Human parainfluenza virus 2 (hPIV2) AF533010; Human parainfluenza virus 3 (hPIV3) Z11575; Human parainfluenza virus 4a (hPIV4a) AB543336; Human parainfluenza virus 4b (hPIV4b) EU627591; J virus (JPV) AY900001; Menangle virus (MenPV) AF326114; Measles virus (MeV) AB016162; Mossman virus (MosPV) AY286409; Mapeura virus (MprPV) EF095490; Mumps virus (MuV) AB000388; Newcastle disease virus (NDV) AF077761; Nipah virus, Bangladesh strain (NiV-B) AY988601; Nipah virus, Malaysian strain (NiV-M) AJ627196; Parainfluenza virus 5 (PIV5) AF052755; Peste-des-petits-ruminants (PPRV) X74443; Porcine rubulavirus (PorPV) BK005918; Rinderpest virus (RPV) Z30697; Salem virus (SalPV) AF237881; Sendai virus (SeV) M19661; Simian virus 41 (SV41) X64275; Tioman virus (TioPV) AF298895; Tupaia paramyxovirus (TupPV) AF079780.

Phylogenetic analysis based on the full length genome sequence and the deduced amino acid sequences of each structural protein confirmed the initial observation that CedPV is most closely related to henipaviruses in the family. A phylogenetic tree based on the deduced sequences of the nucleocapsid protein (N) is presented in Fig. 2. Phylogenetic tree based on whole genome sequences gave similar results (Fig. S4). CedPV is more closely related to HeV and NiV than henipavirus-like sequences detected in African bats [26], [32] as shown in a phylogenetic tree based on the only sequences available of a 550-nt L gene fragment (Fig. S5).

### A phosphoprotein (P) gene lacking RNA editing and coding capacity for the V protein

First discovered for the parainfluenza virus 5 (PIV5, previously known as simian virus 5), almost all members of *Paramyxovirinae* have a P gene which produces multiple proteins through an RNA editing mechanism by addition of non-templated G residues leading to production of N-terminal co-linear proteins from different reading frames downstream from the editing site [3], [33]. These multiple gene products are known to play a key role in antagonizing the innate response of susceptible hosts [3]. A search of CedPV for open reading frames (ORF) in the P gene revealed a 737-aa P protein and a 177-aa C protein, but failed to find the highly conserved, cysteine-rich V ORF present in most other paramyxoviruses. The RNA editing site with the sequence of AAAAGGG, which is absolutely conserved in all known HeV and NiV isolates discovered to date, is also missing from the CedPV P gene sequence. To further verify that there are no multiple mRNAs produced from the CedPV P gene, direct sequencing of P gene transcripts was conducted from CedPV-infected Vero cells using multiple sets of primers generating overlapping fragments covering the entire coding region of the P gene. Each produced uniform trace files indicating a lack of RNA editing activities, which is very different from the mixed peaks generated by HeV and NiV immediately after the editing site (Fig. S7). To our knowledge, CedPV is the first member of *Paramyxovirinae* that lacks both RNA editing and any V-related coding sequence in its P gene. Further investigation is required to exclude the possibility that the P-gene editing in CedPV is cell- or tissue-specific and not present or present at an extremely low level in the current virus-cell system.

### Antigenic relatedness with henipaviruses

**Figure 3:**
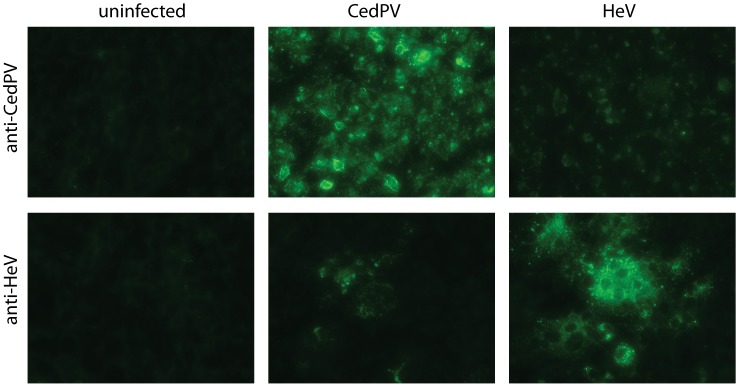
Antigenic cross reactivity between CedPV and HeV. Vero cells infected with CedPV and HeV, respectively, were stained with rabbit sera raised against recombinant N proteins of each virus.

The striking similarity in genome size and organization and the presence of highly conserved protein domains among the N, M and L proteins between CedPV and henipaviruses prompted us to investigate the antigenic relatedness of these viruses. Staining of CedPV- infected Vero cells using rabbit anti-henipavirus antibodies indicated the presence of cross-reactivity. This cross-reactivity was further confirmed in reverse by staining of HeV-infected Vero cells using a rabbit serum raised against a recombinant CedPV N protein (Fig. 3). However, analysis by virus neutralization test using either polyclonal or monoclonal antibodies found that henipavirus-neutralizing antibodies were unable to neutralize CedPV. Similarly, CedPV-neutralizing antibodies obtained in our infection studies (see below) also failed to neutralize either HeV or NiV. It can therefore be concluded that CedPV and henipaviruses share cross-reactive antigenic regions, but not cross-neutralizing epitopes.

### Use of ephrin-B2 as a functional receptor for membrane fusion and entry by CedPV

**Figure 4:**
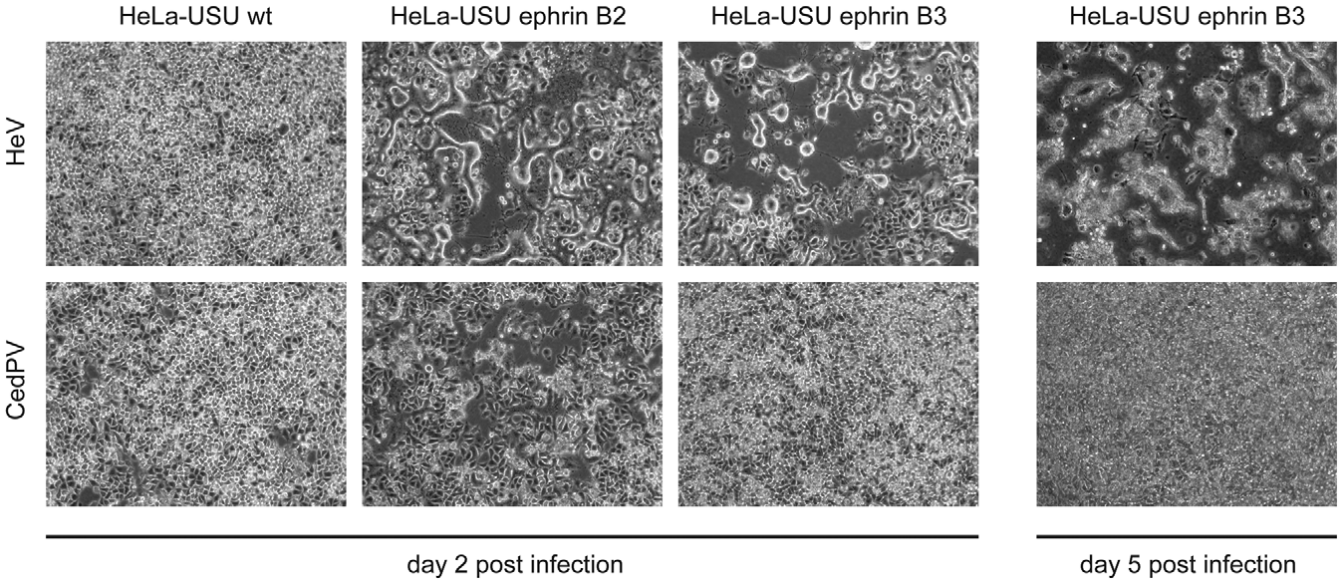
Functional testing of ephrin-B2 and -B3 as an entry receptor for CedPV. Infection of HeLa-USU cells by CedPV in the presence and absence of ephrin gene products. The susceptibility of infection, as an indirect measurement of receptor function, is demonstrated by the formation of syncytial CPE.

To further investigate the relationship between CedPV and recognized henipaviruses, we investigated the use of the henipavirus receptors, the ephrin-B2 and -B3 host cell proteins, as potential receptors for CedPV infection. Our previous studies have demonstrated that the ephrin-B2 and -B3 expression negative HeLa-USU cell line could support henipavirus infection and formation of syncytial CPE only when either the ephrin-B2 or -B3 gene was transiently expressed in the cells [22], [34]. For CedPV, similar observations were made with respect to the ephrin-B2 receptor. As shown in Fig. 4, CedPV failed to infect HeLa-USU, but was able to infect and cause syncytial CPE when the human ephrin-B2 gene was expressed. In contrast, when ephrin-B3 molecule was introduced, there was no evidence of infection.

### Pathogenicity for laboratory mammals

**Figure 5:**
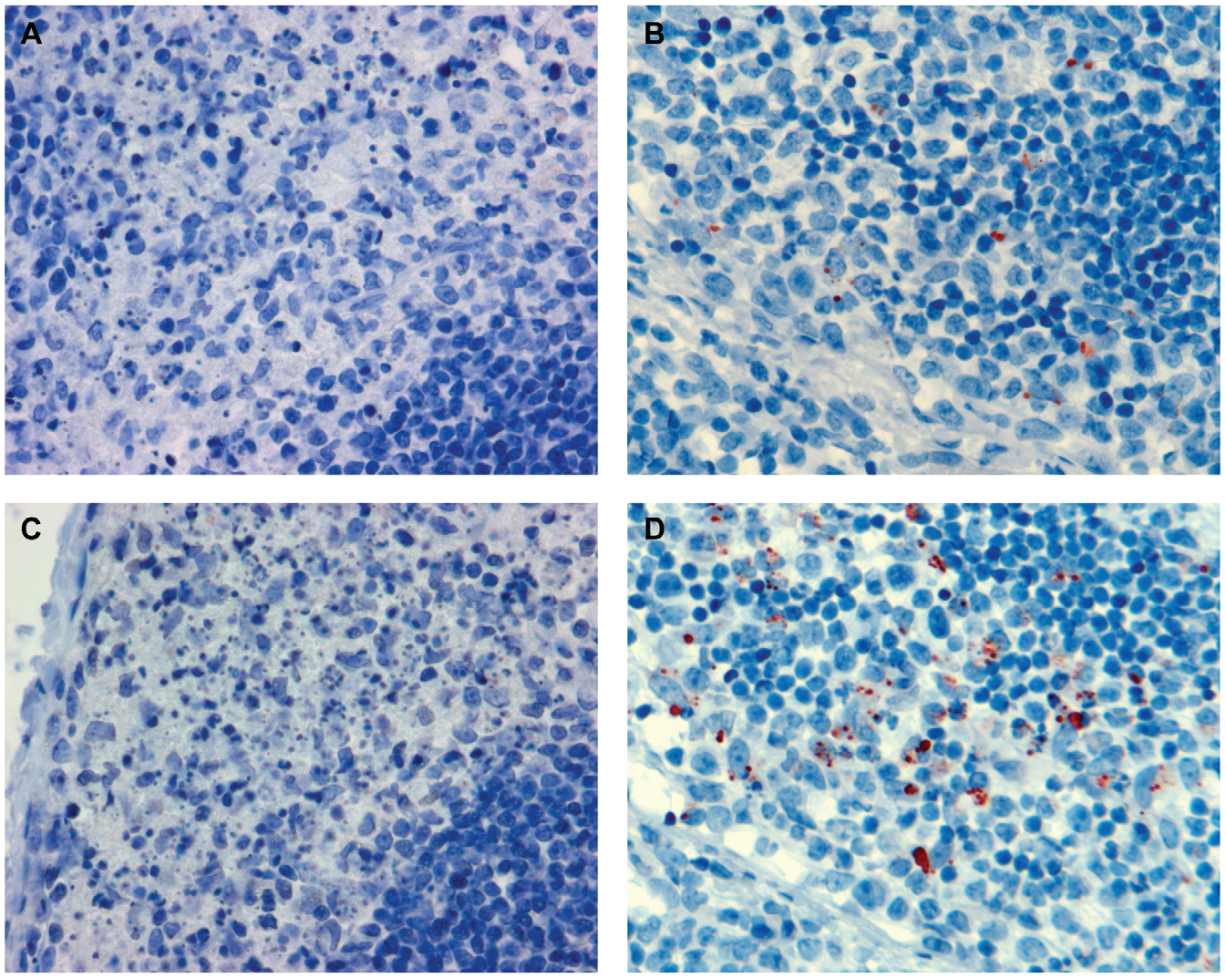
Immunohistochemical analysis of bronchial lymph node of CedPV infected ferrets. Bronchial lymph node of ferret #2, euthanized on day 6 pi, was stained with rabbit antiserum against recombinant N protein of CedPV (**B**) and NiV (**D**), respectively. Bronchial lymph node of an unrelated ferret (infected with influenza H5N1 from another experiment) was used as negative control and stained with the same anti-CedPV (**A**) and anti-NiV (**C**) antisera under identical conditions.

Ferrets, guinea pigs, and mice exhibit differing responses to the previously described henipaviruses HeV and NiV, with ferrets and guinea pigs, but not mice developing severe disease characterized by systemic vasculitis [20], [35], [36], [37], [38]. In contrast, ferrets and guinea pigs exposed to CedPV by, respectively, oronasal and intraperitoneal routes remained clinically well although neutralizing antibody was detected in serum between 10 to 21 days pi (Table 2). Balb-C mice exposed to CedPV by the oronasal route remained clinically well and did not develop neutralizing antibody in serum by day 21 pi. In ferrets electively euthanized at earlier time-points, there was reactive hyperplasia of tonsillar lymphoid tissue, retropharyngeal and bronchial lymph nodes, accompanied by edema and erythrophagocytosis. CedPV antigen was detected in bronchial lymph node of one animal euthanized on day 6 pi, consistent with viral replication in that tissue; cross-reactive immunostaining against anti-NiV N protein antibodies was also noted (Fig. 5). No other significant histological lesions were identified. Viral RNA was detected in selected lymphoid tissues of 3 (of 4) ferrets sampled day 6 to 8 pi, including pharynx, spleen, and retropharyngeal and bronchial lymph nodes, as well as the submandibular lymph node of the ferret euthanized on day 20 pi. This pattern of lymphoid involvement suggests that there may be transient replication in the upper and lower respiratory tracts although CedPV genome was not recovered from nasal washes, oral swabs, pharynx or lung tissue of affected animals. Virus isolation was unsuccessful for all PCR positive tissues.

**Table 2:** Antibody responses in CedPV-infected ferrets and guinea pigs.

Animal #	Days post inoculation	Neutralizing antibody titers	
		CedPV	HeV
**Ferret 1**	0	-ve	-ve
	10	1∶320	-ve
	15	1∶640	-ve
	21	1∶1280	-ve
**Ferret 2**	0	-ve	-ve
	10	1∶320	-ve
	15	1∶640	-ve
	21	1∶1280	-ve
**Guinea pig 1**	0	-ve	-ve
	10	-ve	-ve
	21	1∶80	-ve
**Guinea pig 2**	0	-ve	-ve
	10	-ve	-ve
	21	-ve	-ve
**Guinea pig 3**	0	-ve	-ve
	10	-ve	-ve
	21	-ve	-ve
**Guinea pig 4**	0	-ve	-ve
	10	-ve	-ve
	21	1∶160	-ve

### Induction of IFN responses upon infection

**Figure 6:**
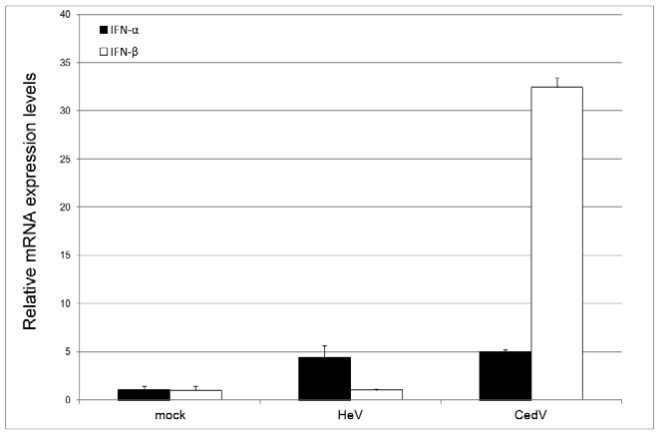
Induction of IFN responses upon henipavirus infection. HeLa cells were infected at an MOI 0.5 for 24 hours. Total RNA was isolated, and quantitative real-time PCR for IFN-α and IFN-β was performed. *n* = 2, with error bars indicating SEM.

As a first step towards the understanding of the pathogenicity difference between CedPV and HeV, we examined the IFN responses in human HeLa cells upon virus infection. As shown in Fig. 6, while the induction of IFN-α was similar in cells infected with HeV or CedPV, there was a significant difference of IFN-β production upon infection by HeV or CedPV, with CedPV-infected cell producing a much higher level of IFN-β.

### Prevalence of neutralizing antibodies in Australian fruit bats

To investigate the CedPV exposure status of pteropid bats in Queensland and potential co-infection (either concurrent or consecutive) of CedPV with HeV, we tested 100 flying fox sera collected previously for other studies for antibody against the two viruses. Due to the cross-reactivity observed above, virus neutralization tests were conducted to obtain more accurate infection data for each virus. Overall, 23% of the sera were CedPV-positive and 37% HeV-positive (Table S1). Co-infection was reflected in 8% of the sera tested.

## Discussion

The emergence of bat-borne zoonotic viruses (including HeV, NiV, Ebola, Marburg, and SARS) has had a significant impact on public health and the global economy during the past few decades. With the rapidly expanding knowledge of virus diversity in bat populations around the world, it is predicted that more bat-borne zoonotic viruses are likely to emerge in the future. The discovery of a novel ebolavirus-like filovirus in Spanish microbats demonstrates that the potential for such spill over events is not limited to Africa or Asia [39]. It is therefore important to enhance our preparedness to counter future outbreaks by conducting active pre-emergence research into surveillance, triggers for cross-species transmission, and the science of identification of potential pathogens.

Henipaviruses represent one of the most important bat-borne pathogens to be discovered in recent history. Although CedPV displays some differences from existing members of the genus *Henipavirus*, we propose that CedPV be classified as a new henipavirus based on the following shared features with known henipaviruses: 1) it is antigenically related to current henipaviruses; 2) its genome size and organization is almost identical to those of HeV and NiV; 3) it has a similar prevalence in flying foxes; and 4) it uses ephrin-B2 as the cell entry receptor.

The lack of cross-neutralization between CedPV and HeV or NiV was not unexpected from the comparative sequence analysis of all the deduced proteins, especially the G protein (see Table 1). It is clear that the genetic relatedness of CedPV with HeV or NiV is much lower than between HeV and NiV. However, the percentage sequence identities of the major viral proteins between CedPV and HeV/NiV are on average at least 10% higher than that between HeV/NiV and any other known paramyxoviruses. Also, there was no antigenic cross-reactivity observed between CedPV and representative viruses of the other paramyxovirus genera in the subfamily *Paramyxovirinae* (Fig. S6).

Like other paramyxoviruses, the P gene of henipaviruses produces multiple proteins which play a key role in viral evasion of host innate immune responses [4], [40], [41]. One of these is the Cys-rich V protein: all members of the subfamily *Paramyxovirinae* produce the V protein with the exception of the human parainfluenza virus 1 (hPIV1). Although a putative RNA editing sequence (AAGAGGG) is present at the expected editing site of the P gene, the hPIV1 RNA polymerase does not produce an edited mRNA of the P gene [42]. There are remnants of the V ORF easily detectable in the hPIV1 P gene although the predicted 68-aa ORF region is interrupted by multiple in-frame stop codons. Of the 7 Cys residues conserved between bovine parainfluenza virus 3 and Sendai virus, four are still present in the non-functional V ORF of hPIV1[42]. In contrast, an extensive ORF and sequence homology search of the CedPV P gene only identified one aa coding region with minimal sequence identity to the V ORFs of HeV and NiV (see Fig. S8). In this region, out of the 9 Cys residues conserved between HeV and NiV V proteins, only 2 are present in the CedPV P gene. Furthermore, the sequence (AGATGAG) upstream from this putative ORF V coding region does not match the consensus RNA editing site. It can therefore be concluded that CedPV is the only member of *Paramyxovirinae* which lacks both the functional V mRNA/protein and the coding capacity for the RNA editing site and ORF V. The evolutionary significance of this finding needs further investigation.

Our *in vitro* study indicated that ephrin B2, but not ephrin B3, was able to restore CedPV infection in the ephrin B2-deficient HeLa cells. While this is highly suggestive that ephrin B2 is the functional entry receptor for CedPV, it should be emphasized that this was not a direct proof that ephrin B2 is the receptor. Further investigation is required to confirm this.

In our preliminary studies, it was shown that CedPV was able to replicate in guinea pigs and ferrets, but failed to cause significant clinical diseases, unlike that of the closely related HeV and NiV. These first infection experiments were conducted with a high dose if virus to establish whether the CedPV could replicate in these animals and determine the degree of any clinical disease. A second experiment was then carried out in ferrets to determine the site of replication and tissue tropism in sequentially sacrificed animals. A lower dose was used to gain better comparison with similar infection experiments using HeV and NiV [18], [35]. Although these initial experimental infection studies indicate that CedPV is less or non-pathogenic in these species, it is possible that CedPV may be pathogenic in other hosts, such as horses. We hypothesize that the lack of a V protein may impact on the pathogenicity. In this regard, it was encouraging to observe that infection of human cells by CedPV induced a much more robust IFN-β response than HeV. Further study is required to dissect the exact molecular mechanism of this observed difference.

Due to the close relationship between CedPV and HeV, it was important to investigate the possibility of co-infection by these two viruses in the Australian bat population. Based on the detection of neutralizing antibodies at 23% for CedPV, 37% for HeV and 8% for both, it can be concluded that the co-infection rate is very close to the theoretical rate of 8.5% (the product of the two independent infection rates). Based on this limited preliminary analysis, it appears that infection of bats by one henipavirus neither prevents nor enhances the likelihood of infection by the other.

In summary, the discovery of another henipavirus in Australian flying foxes highlights the importance of bats as a significant reservoir of potential zoonotic agents and the need to expand our understanding of virus-bat relationships in general. Our future research will be directed at determining whether spill-over of CedPV into other hosts has occurred in the past in Australia, whether CedPV is pathogenic in certain mammalian hosts, and whether CedPV exists in bat populations in geographically diverse regions.

## Materials and Methods

All animal studies were approved by the CSIRO Australian Animal Health Laboratory's Animal Ethics Committee and conducted following the Australian National Health and Medical Research Council Code of Practice for the Care and Use of Animals for Scientific Purposes guidelines for housing and care of laboratory animals.

### Cell culture

Cell lines used this study were Vero (ATCC), HeLa-USU [22], and the *P. alecto* primary cell lines derived from kidney (PaKi), brain (PaBr), (spleen) PaSp and placenta (PaPl) recently established in our group [27]. Cells were grown in Dulbecco's Modified Eagle's Medium Nutrient Mixture F-12 Ham supplemented with double strength antibiotic-antimycotic (Invitrogen), 10 µg/ml ciprofloxacin (MP Biomedicals) and 10% fetal calf serum at 37°C in the presence of 5% CO_2_.

### Urine collection and virus isolation

Urine (approximately 0.5–1 ml) was collected off plastic sheets placed underneath a colony of flying foxes (predominantly *Pteropus alecto* with some *P. Poliocephalus* in the mixed population) in Cedar Grove, South East Queensland, Australia and pooled into 2-ml tubes containing 0.5 ml of viral transport medium (SPGA: a mix of sucrose, phosphate, glutamate and albumin plus penicillin, streptomycin and fungizone). The tubes were temporarily stored on ice after collection and transported to a laboratory in Queensland, frozen at −80°C, and then shipped on dry ice to the CSIRO Australian Animal Health Laboratory (AAHL) in Geelong, Victoria for virus isolation. The samples were thawed at 4°C and centrifuged at 16,000×*g* for 1 min to pellet debris. Urine in the supernatant (approximately 0.5–1 ml) was diluted 1∶10 in cell culture media. The diluted urine was then centrifuged at 1,200×*g* for 5 min and split evenly over Vero, PaKi, PaBr, PaSp and PaPl cell monolayers in 75-cm^2^ tissue culture flasks. The flasks were rocked for 2 h at 37°C, 14 ml of fresh cell culture media was added and then incubated for 7 d at 37°C. The flasks were observed daily for toxicity, contamination, or viral cytopathic effect (CPE).

### Molecular characterization

Cells showing syncytial CPE were screened using published broadly reactive primers [31] for all known paramyxoviruses and a subset of paramyxoviruses. PCR products were gel extracted and cloned into pGEM T-Easy (Promega) to facilitate sequencing using M13 primers. Sequences were obtained and aligned with known paramyxovirus sequences allowing for initial classification.

Whole genome sequence was determined using a combination of 454 sequencing [43] and conventional Sanger sequencing. Virions from tissue culture supernatant were collected by centrifugation at 30,000×*g* for 60 min and resuspended in 140 µl of PBS and mixed with 560 µl of freshly made AVL for RNA extraction using QIAamp Viral RNA mini kit (Qiagen). Synthesis of cDNA and random amplification was conducted using a modification of a published procedure [44]. Briefly, cDNA synthesis was performed using a random octomer-linked to a 17-mer defined primer sequence: (5′-GTTTCCCAGTAGGTCTCNNN NNNNN-3′) and SuperScript III Reverse Transcriptase (Invitrogen). 8 µl of ds-cDNA was amplified in 200 µl PCR reactions with hot-start Taq polymerase enzyme (Promega) and 5′-A*G*C*A*C TGTAGGTTTCCCAGTAGGTCTC-3′ (where * denotes thiol modifications) as amplification primers for 40 cycles of 95°C/1 min, 48°C/1 min, 72°C/1 min after an initial denaturation step of 5 min at 95°C and followed by purification with the QIAquick PCR purification kit (Qiagen). Sample preparation for Roche 454 sequencing (454 Life Sciences Branford, CT, USA) was according to their Titanium series manuals, Rapid Library Preparation and emPCR Lib-L SV.

To obtain an accurate CedPV genome sequence, 454 generated data (after removing low quality, ambiguous and adapter sequences) was analysed by both de novo assembly and read mapping of raw reads onto the CedPV draft genome sequence derived from Sanger sequencing. For 454 read mapping, SNPs and DIPs generated with the CLC software were manually assessed for accuracy by visualising the mapped raw reads (random PCR errors are obvious compared to real SNPs and DIPs especially when read coverage is deep). Consensus sequences for both 454 de novo and read mapping assembly methods were then compared to the Sanger sequence with the latter used to resolve conflicts within the low coverage regions as well as to resolve 454 homopolymer errors.

Sequences of genome termini were determined by 3′- and 5′-RACE using a protocol previously published by our group [45]. Briefly, approximately 100 ng of RNA was ligated with adaptor DT88 (see reference for sequence information) using T4 RNA ligase (Promega) followed by cDNA synthesis using the SuperScript III RT kit (Invitrogen) and an adaptor-specific primer, DT89. PCR amplification was then carried out using DT89 and one or more genome-specific primers. PCR products were sequenced directly using either DT89 or genome specific primers by an in-house service group on the ABI Sequencer 3100.

### Sequence analysis

The CLC Genomics Workbench v4.5.1 (CLC Inc, Aarhus, Denmark) was used to trim 454 adapter and cDNA/PCR primer sequences, to remove low quality, ambiguous and small reads <15 bp and to perform *de novo* and read mapping assemblies all with default parameters. Clone Manager Professional ver 9.11 (Scientific and Educational Software, Cary, NC, USA) was used to join overlapping contigs generated by *de novo* assembly. Phylogenetic trees were constructed by using the neighbor-joining algorithm with bootstrap values determined by 1,000 replicates in the MEGA4 software package [46].

### Real time PCR

Quantitative PCR assays (qPCR) were established based on CedPV-specific sequences obtained from the high throughput sequencing. A TaqMan assay on the P gene was developed and used for all subsequent studies. The sequences of the primer/probe are as follows: forward primer, 5′-TGCAT TGAGC GAACC CATAT AC; reverse primer, 5′-GCACG CTTCT TGACA GAGTT GT; probe, 5′-TCCCG AGAAA CCCTC TGTGT TTGA-MGB.

### Production of recombinant antigen and rabbit sera

The coding region for the CedPV N protein was amplified by PCR with a pair of primers flanked by *Asc*I (5′ end) and *Not*I (3′ end) sites for cloning into our previously described GST-fusion expression vector [47]. The expression and purification by gel elution was conducted as previously described [48]. For antibody production, purified protein was injected subcutaneously into 4 different sites of 2 adult (at a dose of 100 µg per animal) New Zealand white female rabbits at days 0 and 27. The CSIRO's triple adjuvant [49] was used for the immunization. Animals were checked for specific antibodies after days 5 and 42 and euthanized at day 69 for the final blood collection.

### Antibody tests

For immunofluorescence antibody test, Vero cell monolayers were prepared in 8-well chamber slides by seeding at a concentration of 30,000 cells/well in 300 µl of cell media and incubating over night at 37°C. The cell monolayers were infected with an MOI of 0.01 of CedPV, HeV or NiV and fixed with 100% ice-cold methanol at 24 h post-infection. The chamber slides were blocked with 100 µl/well of 1%BSA in PBS for 30 min at 37°C before adding 50 µl/well of rabbit sera against CedPV N or NiV N diluted 1∶1000. After incubation at 37°C for 30 min, the slides were washed three times in PBS-T and incubated with 50 µl/well of anti-rabbit 488 Alexafluore conjugate (Invitrogen) diluted 1∶1000 at 37°C for 30 min. The slides were then washed three times in PBS-T and mounted in 50% glycerol/PBS for observation under a fluorescence microscope.

For virus neutralization test, serial two-fold dilutions of sera were prepared in duplicate in a 96-well tissue culture plate in 50 µl cell media (Minimal Essential Medium containing Earle's salts and supplemented with 2 mM glutamine, antibiotic-antimycotic and 10% fetal calf serum). An equal volume containing 200 TCID_50_ of target virus was added and the virus-sera mix incubated for 30 min at 37°C in a humidified 5% CO_2_ incubator. 100 µl of Vero cell suspension containing 2×10^5^ cells/ml was added and the plate incubated at 37°C in a humidified 5% CO_2_ incubator. After 4 days, the plate was examined for viral CPE. The highest serum dilution generating complete inhibition of CPE is defined as the final neutralizing titer.

### Testing of receptor specificity

Human ephrin B2 and B3 genes were cloned into pQCXIH (Clontech) and the resulting plasmids packaged into retrovirus particles in the GP2–293 packaging cell line (Clontech) and pseudotyped with vesicular stomatitis virus G glycoprotein (VSV-G) following the manufacturer's instructions. HeLa-USU cell line [22] was infected with the VSV-G pseudotyped retrovirus particles in the presence of 1 µg/ml polybrene (Sigma). 8 h post infection, the medium was changed and the cells were allowed to recover for 24 h, allowing time for the retroviral insert to be incorporated into the cell genome and for expression of the hygromycin resistance gene. 24 h post infection, cells transformed by the retrovirus were selected for by the addition of 200 µg/ml hygromycin in the media. Stocks of cells that were resistant to hygromycin were prepared and frozen. HeLa-USU and ephrin-expressing HeLa-USU cells were seeded in 6-well tissue culture plates at a density of 250,000 cells/well overnight. The viruses (HeV and CedPV) were diluted to give an MOI of 0.01 and inoculated into the wells. The cell monolayers were examined daily for syncytial CPE.

### Animal infection studies

Animal studies were carried out in the BSL4 animal facility at AAHL. Ferrets, guinea pigs and mice were used on the basis of their known and varying responses to exposure to other henipaviruses.

Firstly, 2×10^6^ TCID_50_/ml CedPV passaged twice in bat PaKi cells was administered to 2 male ferrets (1 ml oronasally); 4 female guinea pigs (1 ml intraperitoneally); and 5 female Balb-C mice (50 µl oronasally). Guinea pigs and mice were implanted with temperature sensing microchips (LifeChip Bio-thermo, Destron Fearing) and weighed daily. Ferret rectal temperature and weight was recorded at sampling times. Animals were observed daily for clinical signs of illness and were euthanized at 21 d post-inoculation. Sera were collected on days 10, 15 and 21 to test for neutralizing antibody against CedPV.

Secondly, on the basis of asymptomatic seroconversion to CedPV noted in ferrets in the first study, 7 further female ferrets were exposed by the oronasal route to a lower dose of 3×10^3^ TCID_50_. Two animals were euthanized on each of days 6, 8 and 10 post-inoculation and one on day 20. Nasal washes, oral swabs, and rectal swabs were collected on days 2, 4, 6, 8 and 10 and urine was sampled on the day of euthanazia; each specimen was assessed for CedPV genome. A wide range of tissue samples were collected at post mortem examination and assessed by routine histology, immunohistochemistry (using rabbit antibodies raised against recombinant CedPV and NiV N proteins, respectively), qPCR (see above) and virus isolation using reagents and procedures previously established in our group [16].

### Determination of IFN responses

HeLa cells were infected with Hendra and Cedar viruses at an MOI 0.5 for 24 hours, at which time total cellular RNA was extracted and IFN-α and IFN-β mRNA levels were quantified by real-time PCR using Power SYBR Green RNA-to-CT 1-Step Kit (Applied Biosystems). Primers were as previously described [50].

### Serological survey

Sera from 100 flying foxes collected during 2003–2005 from Queensland, Australia were screened for neutralizing antibodies to CedPV. Virus neutralization test was conducted as described above (antibody tests). All serum samples were tested at a dilution of 1∶20.

## Supporting Information

Figure S1 **Cytopathic effect (CPE) observed in Paki cells.** This is the original syncytial CPE seen in Paki cells 5 days post inoculation.
(TIF)

Figure S2 **Map location of the sampling site, Cedar Grove, in southeast Queensland.** The location of the index Hendra virus outbreak in 1994 is shown by a green dot while the sampling site of the current study is marked by a red star.
(TIF)

Figure S3 **Comparison of genomic features among different henipaviruses.** (A) Alignment of leader and trailer sequences (antigenome sequences shown). (B) Sequences of intergenic regions (IGR) and transcriptional start and stop sties of CedPV in comparison with those of HeV and NiV.
(DOCX)

Figure S4 **Phylogenetic trees of viruses in the subfamily** ***Paramyxovirinae*** **based on whole genome sequence.**
(TIF)

Figure S5 **Phylogenetic trees of viruses in the subfamily** ***Paramyxovirinae*** **based on a 550-nt region of the L-gene.**
(TIF)

Figure S6 **Sequencing trace files for the editing site of P genes for HeV and NiV in comparison to a putative editing site of the CedPV P gene.** Trace files showing editing of the HeV and NiV P gene (indicated by the * sign) and lack of editing in CedPV P gene mRNA in infected cells. Sequencing of PCR products covering all potential editing sites in the P gene of CedPV did not reveal any RNA editing activity. A representative potential editing site (see Fig. S8) of the CedPV P gene is shown.
(TIF)

Figure S7 **Determination of antigenic cross reactivity with other paramyxoviruses.** Shown here are IFAT conducted with anti-CedPV serum on Vero cells infected with J paramyxovirus (JPV), Rinderpest virus (RPV), Sendai virus (SeV), Menangle virus (MenPV) and CedPV, respectively. Mock infected cell monolayer was included as a negative control.
(TIF)

Figure S8 **Sequence alignments of putative V ORF (A) and mRNA editing site (B) among HeV, NiV and CedPV.**
(DOCX)

Table S1 **Prevalence of neutralizing antibodies to CedPV and HeV in Australian flying foxes.**
(DOCX)
