# Supplementary figures and images for: Cedar Virus: A Novel Henipavirus Isolated from Australian Bats
Source: PLoS Pathog. 2012 Aug 2;8(8):e1002836. doi: 10.1371/journal.ppat.1002836 (PMC3410871; doi:10.1371/journal.ppat.1002836)

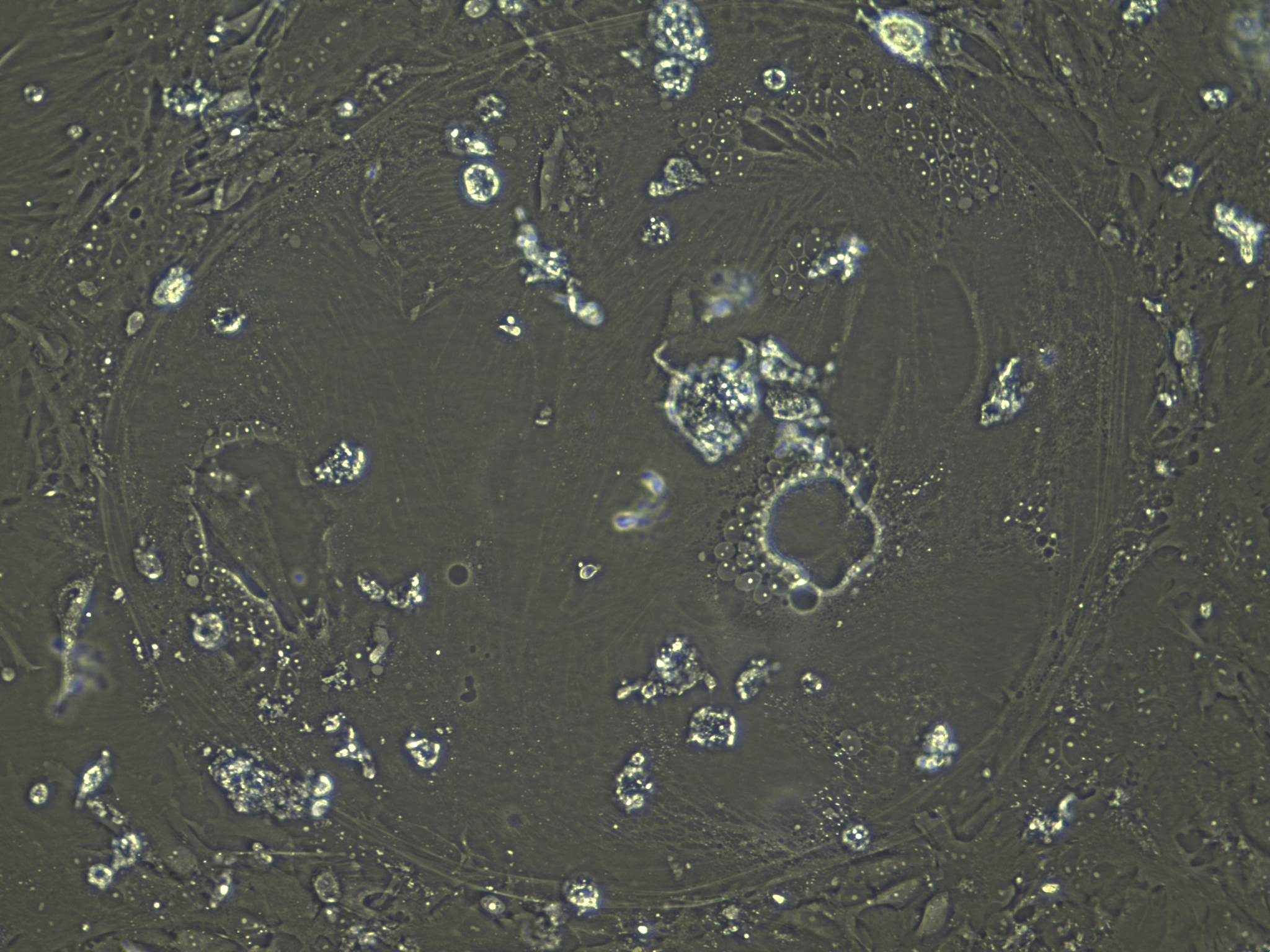

Supplement: Figure S1 — Cytopathic effect (CPE) observed in Paki cells. This is the original syncytial CPE seen in Paki cells 5 days post inoculation. (TIF) [file ppat.1002836.s001.tif]

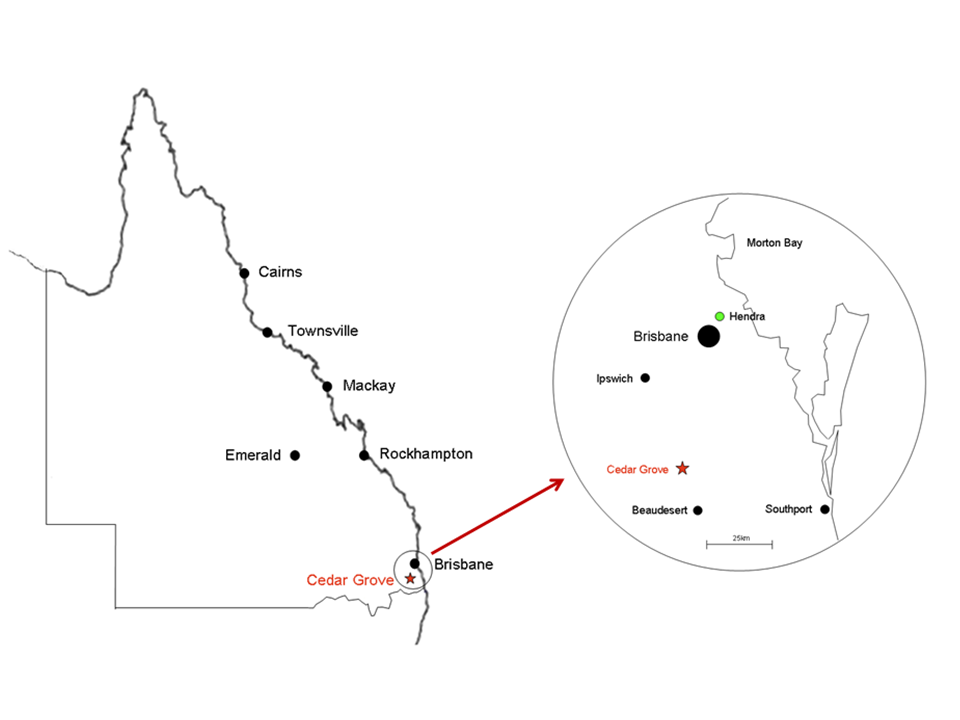

Supplement: Figure S2 — Map location of the sampling site, Cedar Grove, in southeast Queensland. The location of the index Hendra virus outbreak in 1994 is shown by a green dot while the sampling site of the current study is marked by a red star. (TIF) [file ppat.1002836.s002.tif]

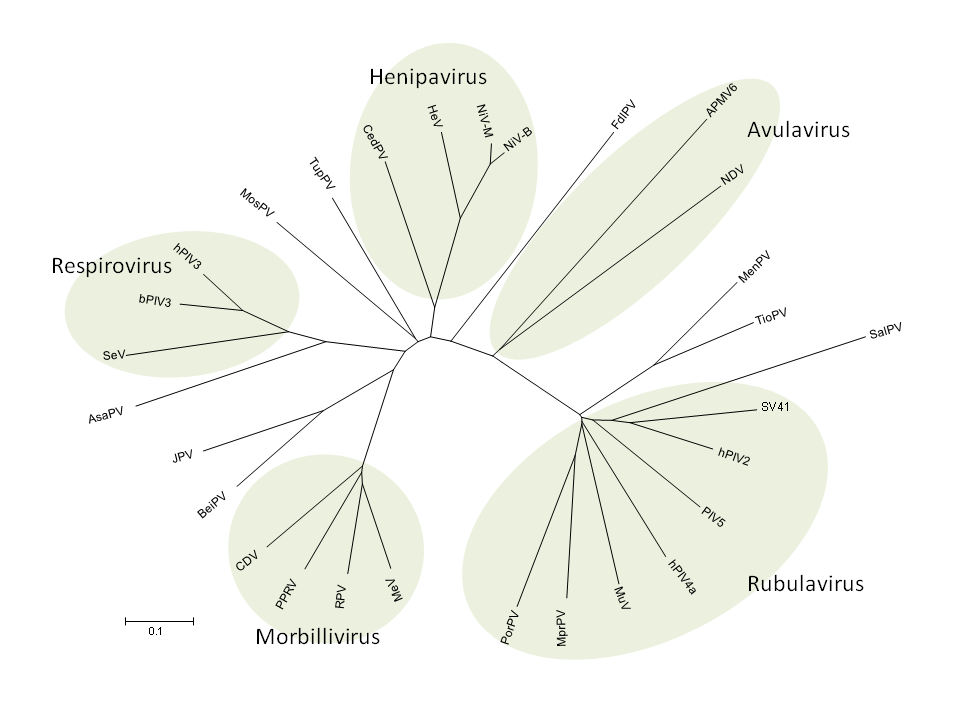

Supplement: Figure S4 — Phylogenetic trees of viruses in the subfamily Paramyxovirinae based on whole genome sequence. (TIF) [file ppat.1002836.s004.tif]

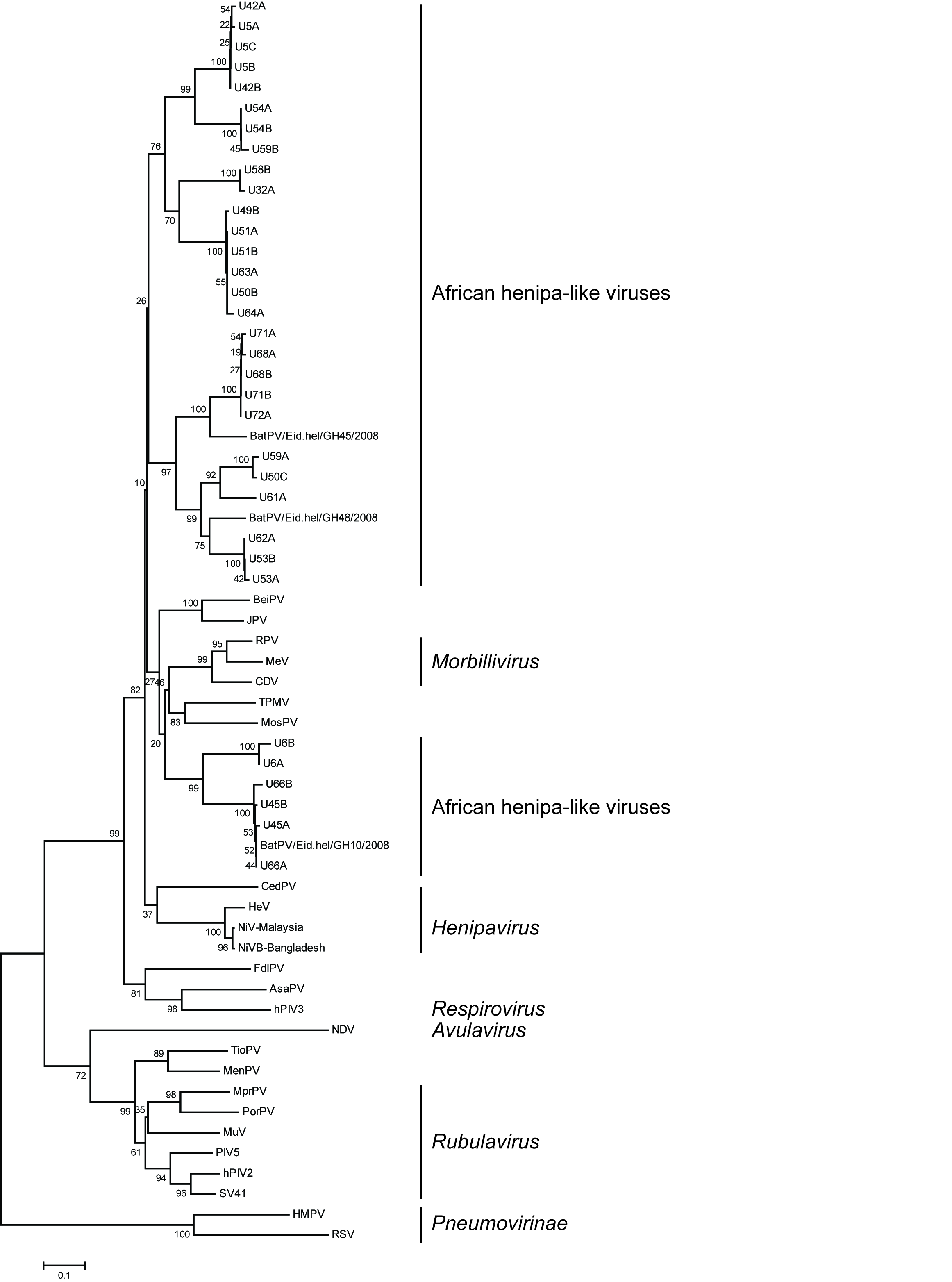

Supplement: Figure S5 — Phylogenetic trees of viruses in the subfamily Paramyxovirinae based on a 550-nt region of the L-gene. (TIF) [file ppat.1002836.s005.tif]

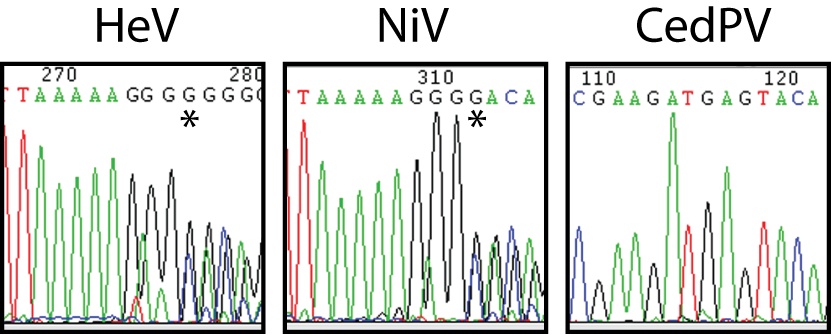

Supplement: Figure S6 — Sequencing trace files for the editing site of P genes for HeV and NiV in comparison to a putative editing site of the CedPV P gene. Trace files showing editing of the HeV and NiV P gene (indicated by the * sign) and lack of editing in CedPV P gene mRNA in infected cells. Sequencing of PCR products covering all potential editing sites in the P gene of CedPV did not reveal any RNA editing activity. A representative potential editing site (see Fig. S8) of the CedPV P gene is shown. (TIF) [file ppat.1002836.s006.tif]

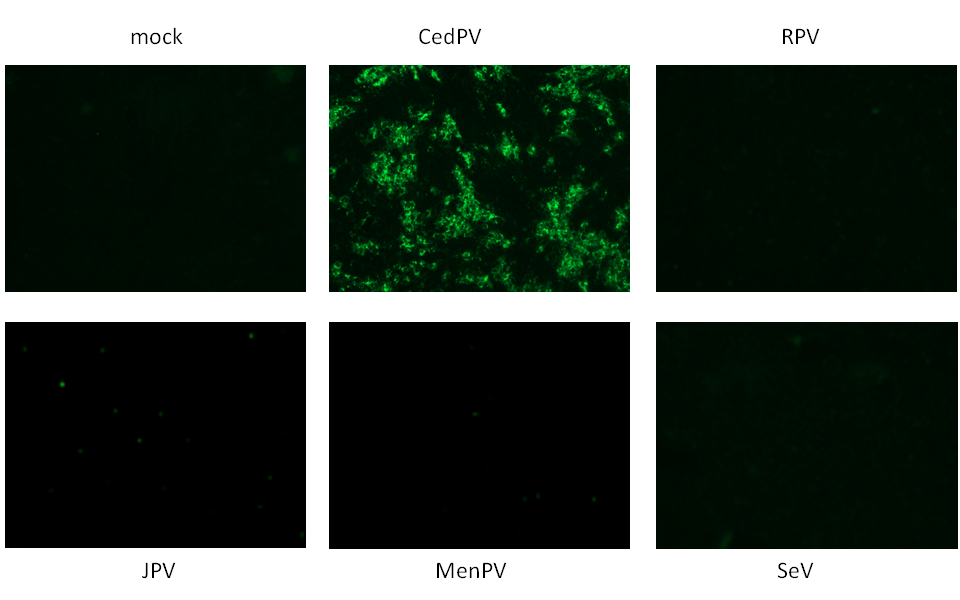

Supplement: Figure S7 — Determination of antigenic cross reactivity with other paramyxoviruses. Shown here are IFAT conducted with anti-CedPV serum on Vero cells infected with J paramyxovirus (JPV), Rinderpest virus (RPV), Sendai virus (SeV), Menangle virus (MenPV) and CedPV, respectively. Mock infected cell monolayer was included as a negative control. (TIF) [file ppat.1002836.s007.tif]
