# Supplementary material for: Cedar Virus: A Novel Henipavirus Isolated from Australian Bats
Source: PLoS Pathog. 2012 Aug 2;8(8):e1002836. doi: 10.1371/journal.ppat.1002836 (PMC3410871; doi:10.1371/journal.ppat.1002836)
Supplement: Figure S3 — Comparison of genomic features among different henipaviruses. (A) Alignment of leader and trailer sequences (antigenome sequences shown). (B) Sequences of intergenic regions (IGR) and transcriptional start and stop sties of CedPV in comparison with those of HeV and NiV. (DOCX) [file ppat.1002836.s003.docx]

**A.**

CedPV 5’-ACCAGAAAAAGG . . . . . CCTTTTTTAGGA-3’

HeV 5’-ACCGAACAAGGG . . . . . CCCTTGTTCGGA-3’

NiV 5’-ACCAAACAAGGG . . . . . CCCTTGTTCGGA-3’

**B.**

**Genes Gene Start IGR Gene Stop**

/N CTT AGGATCCCGG

N/P TTACAAAAAA CTT AGGATCCAAG

P/M TTAGAAAAAA CTT AGGATCCCAG

M/F TTAAGAAAAA CTT AGGATCCCAG

F/G TTAAATAAAA CTT AGGATCCCAG

G/L TTAAAGAAAA CTT AGGATCCCAG

L/ TTAAAGAAAA CTT

**Consensus**

CedPV TTAvrdAAAA CTT AGGATCCmrG

HeV TTAmrAAAAA CTT AGGAnmCArG

NiV TwAwrAAAAA CTT AGGAnmCArG
