# Supplementary material for: Cedar Virus: A Novel Henipavirus Isolated from Australian Bats
Source: PLoS Pathog. 2012 Aug 2;8(8):e1002836. doi: 10.1371/journal.ppat.1002836 (PMC3410871; doi:10.1371/journal.ppat.1002836)
Supplement: Figure S8 — Sequence alignments of putative V ORF (A) and mRNA editing site (B) among HeV, NiV and CedPV. (DOCX) [file ppat.1002836.s008.docx]

**A.**

**# # # # # # # ##**

**HeV-V HRREVSICWDGRRAWVEEWCNPVCSRITPQPRKQECYCGECPTECSQCCHEE***

**NiV-V HRREISICWDGKRAWVEEWCNPACSRITPLPRRQECQCGECPIECSHCCEGN***

**:.:. ::::: : .:. .:: :**

**CedPV-V? YRKEIDICWDGNGVWTNEYWVRRSTRIR*TKG*I*SRC*PNVSV*PRGPGG***

**: Identical**

**. Conserved**

**# Identical C residues in HeV and NiV**

*** In-frame stop codons**

**B.**

**HeV AAAAGGG**

**NiV AAAAGGG**

**CedPV** **AGATGAG**
