# Supplementary material for: Cedar Virus: A Novel Henipavirus Isolated from Australian Bats
Source: PLoS Pathog. 2012 Aug 2;8(8):e1002836. doi: 10.1371/journal.ppat.1002836 (PMC3410871; doi:10.1371/journal.ppat.1002836)
Supplement: Table S1 — Prevalence of neutralizing antibodies to CedPV and HeV in Australian flying foxes. (DOCX) [file ppat.1002836.s009.docx]

| **Sample #** | **CedPV** | **HeV** | **Sample #** | **CedPV** | **HeV** |
| --- | --- | --- | --- | --- | --- |
| 1 | - | + | 51 | + | - |
| 2 | - | + | 52 | + | - |
| 3 | - | - | 53 | + | + |
| 4 | + | - | 54 | + | - |
| 5 | - | + | 55 | - | + |
| 6 | - | - | 56 | - | - |
| 7 | - | + | 57 | + | - |
| 8 | - | - | 58 | - | - |
| 9 | - | + | 59 | - | + |
| 0 | - | - | 60 | - | - |
| 11 | - | + | 61 | - | - |
| 12 | - | + | 62 | + | + |
| 13 | + | + | 63 | - | - |
| 14 | - | - | 64 | + | + |
| 15 | - | + | 65 | - | - |
| 16 | - | - | 66 | - | - |
| 17 | - | - | 67 | - | + |
| 18 | - | + | 68 | - | - |
| 19 | + | - | 69 | - | + |
| 20 | - | + | 70 | + | + |
| 21 | - | + | 71 | - | - |
| 22 | - | - | 72 | - | + |
| 23 | - | + | 73 | - | + |
| 24 | - | - | 74 | - | + |
| 25 | - | - | 75 | - | + |
| 26 | - | + | 76 | - | - |
| 27 | - | + | 77 | - | + |
| 28 | + | - | 78 | - | - |
| 29 | + | + | 79 | - | - |
| 30 | - | - | 80 | - | - |
| 31 | - | - | 81 | - | - |
| 32 | - | - | 82 | - | - |
| 33 | - | - | 83 | + | + |
| 34 | + | + | 84 | - | + |
| 35 | - | + | 85 | - | - |
| 36 | - | - | 86 | + | - |
| 37 | - | - | 87 | - | - |
| 38 | - | - | 88 | - | - |
| 39 | - | - | 89 | - | - |
| 40 | + | + | 90 | - | - |
| 41 | + | - | 91 | - | - |
| 42 | + | - | 92 | - | - |
| 43 | + | - | 93 | - | - |
| 44 | - | + | 94 | - | - |
| 45 | - | - | 95 | - | - |
| 46 | + | + | 96 | - | - |
| 47 | - | - | 97 | - | - |
| 48 | - | - | 98 | - | - |
| 49 | + | - | 99 | - | - |
| 50 | + | + | 100 | - | - |
| **Total positive** | | | | **23/100** | **37/100** |

**Table S1.** Prevalence of neutralizing antibodies to CedPV and HeV in Australian flying foxes

(all sera were tested at a dilution of 1:20)
